# The Morphology, Structure, Mechanical Properties and Biocompatibility of Nanotubular Titania Coatings before and after Autoclaving Process

**DOI:** 10.3390/jcm8020272

**Published:** 2019-02-23

**Authors:** Aleksandra Radtke, Michalina Ehlert, Tomasz Jędrzejewski, Michał Bartmański

**Affiliations:** 1 Faculty of Chemistry, Nicolaus Copernicus University in Toruń, Gagarina 7, 87-100 Toruń, Poland; m.ehlert@doktorant.umk.pl; 2 Nano-implant Ltd., Gagarina 5/102, 87-100 Toruń, Poland; 3 Faculty of Biology and Environmental Protection, Nicolaus Copernicus University in Toruń, Lwowska 1, 87-100 Toruń, Poland; tomaszj@umk.pl; 4 Faculty of Mechanical Engineering, Gdańsk University of Technology, Gabriela Narutowicza 11/12, 80-233 Gdańsk, Poland; michal.bartmanski@pg.edu.pl

**Keywords:** titanium dioxide, nanotubes, autoclaving, titanium alloy, biocompatibility, wettability, mechanical properties

## Abstract

The autoclaving process is one of the sterilization procedures of implantable devices. Therefore, it is important to assess the impact of hot steam at high pressure on the morphology, structure, and properties of implants modified by nanocomposite coatings. In our works, we focused on studies on amorphous titania nanotubes produced by titanium alloy (Ti6Al4V) electrochemical oxidation in the potential range 5–60 V. Half of the samples were drying in argon stream at room temperature, and the second ones were drying additionally with the use of immersion in acetone and drying at 396 K. Samples were subjected to autoclaving and after sterilization they were structurally and morphologically characterized using Raman spectroscopy, diffuse reflectance infrared Fourier transform spectroscopy (DRIFT) and scanning electron microscopy (SEM). They were characterized in terms of wettability, mechanical properties, and biocompatibility. Obtained results proved that the autoclaving of amorphous titania nanotube coatings produced at lower potentials (5–15 V) does not affect their morphology and structure regardless of the drying method before autoclaving. Nanotubular coatings produced using higher potentials (20–60 V) require removal of adsorbed water particles from their surface. Otherwise, autoclaving leads to the destruction of the architecture of nanotubular coatings, which is associated with the changing of their mechanical and biointegration properties.

## 1. Introduction

Titanium and its alloys (Ti6Al4V, Ti5Al25Fe, and Ti6Al7Nb) are commonly used as biomaterials for orthopedic, dental, or neurosurgery applications [1,2,3]. This choice is determined by the beneficial properties of titanium, which is characterized by the excellent corrosion resistance, light weight, high strength, chemical stability, and modulus of elasticity much closer to that one of bone, compared to other metals. Unfortunately, its surface does not form a direct connection with the living bone [3,4,5]. And the most significant factor influencing the efficiency of implantology is the interaction between the body cells and the implanted artificial biomaterial [4,5,6]. In a wide range of clinical applications, implant rejections still occur mainly due to osseointegration defects and infection [7,8]. Many techniques are used to modify the surface topography of implants in order to improve cell adhesion and osteogenic differentiation on them. It has been concluded that the surface roughness is conducive to the osseointegration process, influences the adhesion of osteoblasts, increases their enzymatic activity and determines the amount and type of proteins they synthesize [6,7,9,10,11]. The appropriate porosity of the implant is ensured, by scaffolds manufactured on its surface, which are characterized by the specific structure, morphology, and wettability [12,13]. According to previous reports, the fabrication of the titanium dioxide nanotubes coating (TNT) on the surface of titanium or titanium alloys implants, improves the adhesion and proliferation of osteoblast cells, and promotes the faster growth of bone and vascular tissue [14,15]. TNT coatings on the surfaces of medical devices, fabricated of titanium or titanium alloys, are commonly produced by their electrochemical anodization [5,7,16,17,18,19,20]. The main advantages of this technique are: the mild processing conditions, low costs, possibilities of large-scale production, and, which is the most important, very good control of structural and morphological properties [16,21]. By changing the type and composition of the electrolyte solution, the anodizing voltage, the temperature and duration of anodizing, it is possible to control the diameter and length of the nanotubes [17,21]. For medical applications, it is particularly important to control the diameters of the produced tubes, as their size influences on the coating biocompatibility degree. Considering earlier reports it should be noted that TNT coatings, which consist of nanotubes with different tube sizes (30–100 nm) could enhance osteoblast cell functions [22,23]. Park et al. suggest something opposite - that nanotubes of smaller sizes than 15 nm strongly enhance cell activities and cell functions deteriorate with increasing tube sizes [24]. Results of our works confirm this suggestion. However, this applies to TNT coatings consisted of nanotubes with 25–35 diameters [25]. Zaho et al. noted that the observed divergences might be caused by the used sterilization method and its possible influence on morphology, structure and the biocompatibility of titania nanotubes [26]. Among the sterilization procedures, which are commonly applied in the clinical practice, i.e. gamma radiation, plasma sterilization, ethylene oxide (EO) sterilization, autoclaving, ultraviolent (UV) irradiation, and ethanol immersion [26,27,28,29,30]—autoclaving is the one, we have used in our research. In this method, the medical device’s surface is treated by the hot steam under increased pressure. In the case of implants coated with bioactive TNT layer, such treatment may lead to changes in their surface morphology, structure, mechanical properties, and biocompatibility. 

In our works, we have focused on studies on autoclaving procedure influence on the surface architecture rearrangement, mechanical properties and biointegration of nanotubular systems produced via anodic oxidation of titanium alloy at different potentials (5–60 V). Despite intense works on the application of TNT coatings in clinical practice as well as the wide use of autoclaving as the main sterilization procedure, above-mentioned issues have not been sufficiently investigated. Therefore, the results discussed in this paper can be important both for the design and fabrication of Ti6Al4V implants coated with bioactive coating and also for their practical use for clinicians.

## 2. Experimental Section

### 2.1. Synthesis and Characterization of Studied Coatings

**Scheme 1:**
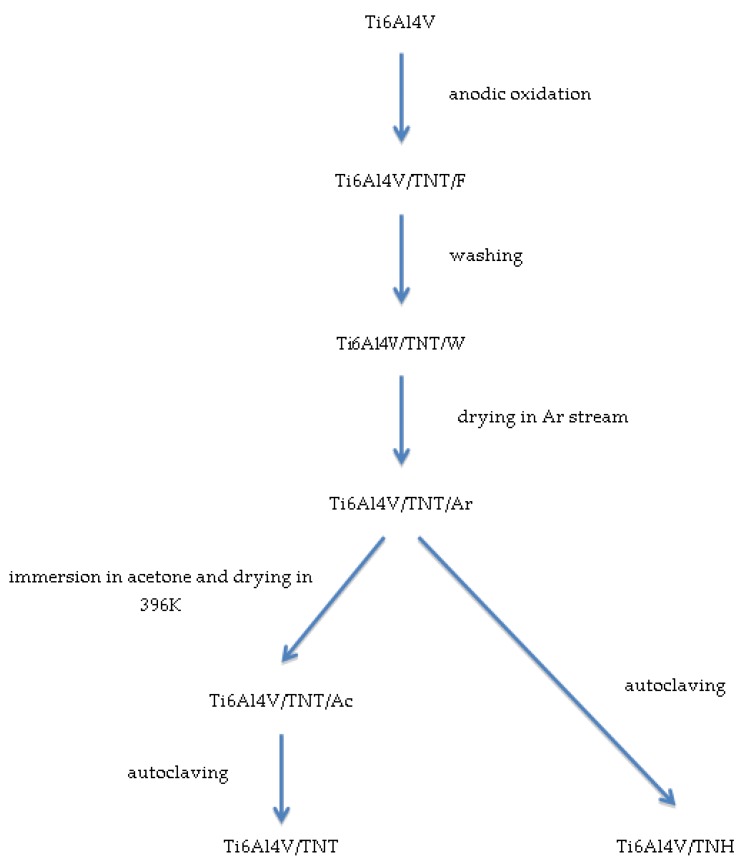
The scheme of experimental procedure applied in the preparation of the samples.

Scheme 1 presents the experimental procedure applied in our studies. The electrochemical anodization of Ti6Al4V foil samples (Ti6Al4V, grade 5, 99.7% purity, 0.20 mm thick (Strem Chemicals, Inc., Bischheim, France), 5 mm × 50 mm strips) was carried out in accordance with the previously described methodology [25]. The uniform TNT coatings were produced using the following potentials: 5, 10, 15, and 20–60 V (every 10V) at room temperature (anodization time *t* = 30 min). After the anodization, all produced nanotubular systems (Ti6Al4V/TNT/F samples; F-freshly obtained) were washed 10 minutes in an ultrasonic bath in deionized water (Ti6Al4V/TNT/W samples; W-washed) and then their surfaces were drying in a stream of argon at room temperature (Ti6Al4V/TNT/Ar samples; Ar-dried in stream of argon). 

Due to the fact that the fabricated TiO_2_ nanotubes could still contain water molecules, adsorbed inside of tubes, which were not removed by the use of the Ar stream, half of the samples were subjected to an additional drying process. These samples were immersed in acetone for 10 min in ultrasonic bath and then were drying at 396 K for 1 h. All produced samples were then autoclaved using IS YESON YS-18L autoclave (Ningbo Haishu Yeson Medical Device Co. Ltd, Zhejiang, China) at 396 K, using *p* = 120 kPa, *t* = 30 min. The surface morphology of studies samples, at every stage of the experimental procedure, was studied, using Quanta scanning electron microscope with field emission (SEM, Quanta 3D FEG, FEI Company, Huston, TX, USA). The structure of the produced coatings was studied using Raman spectroscopy (RamanMicro 200 PerkinElmer (PerkinElmer Inc., Waltham, MA, USA) (λ = 785 nm)) and diffuse reflectance infrared Fourier transform spectroscopy (DRIFT, Spectrum2000, PerkinElmer Inc., Waltham, MA, USA). The wettability and surface free energy of the produced titania nanocoatings were determined using earlier described methods [25,31]. The contact angle was measured using a goniometer (DSA 10 Krüss GmbH, Hamburg, Germany) with drop shape analysis software (ADVANCE). Each measurement was carried out three times.

### 2.2. Topography and Mechanical Properties of Studied Coatings

Surface topographies were examined by means of atomic force microscopy (AFM, NaniteAFM, Nanosurf AG, Liestal, Switzerland) using a contactless module with a force of 20 nN in the 50 × 50 μm area. Hardness tests and Young modulus measurements were carried out using a nanoindenter (NanoTest Vantage, Micro Materials Ltd., Wrexham, UK) with a pyramidal, diamond, three-sided Berkovich indenter, at an apical angle of 124.4°. Hardness tests were performed for loads of 10 mN. The time of load increase from the zero value to the maximum load 10 mN was 15 s. Indentation were performed at one cycle with 5 s dwell at maximum load. Hardness (H), reduced Young’s modulus (E_r_), and Young’s modulus values were determined using the Oliver-Pharr method based on the NanoTest results analysis program. In order to convert the reduced Young’s modulus into Young’s modulus, a Poisson coefficient of 0.25 was assumed for the coatings.

Tests of coatings adhesion were made using nanoindenter (NanoTest Vantage, Micro Materials Ltd., Wrexham, UK) and the Berkovich indenter, as in the case of the nanoindentation tests.

The parameters of scratch tests were as follows: scratch load 0 to 500 mN, loading rate 3.3 mN/s, scan velocity 3 μm/s, and scan length 500 μm. Based on the dependence of the friction force (F_t_) on the normal force (F_n_) in the program for the analysis of NanoTest results, the values of critical friction force (L_f_) and critical force (L_c_), which caused the separation of the layer from the substrate, were determined.

### 2.3. Analysis of Studied Coatings Biointegration Properties

#### 2.3.1. Cell Culture

Human osteoblast-like MG-63 cells (European Collection of Cell Cultures, Salisbury, UK) were plated in a 25 cm^2^ cell culture flask (Corning, Corning, NY, USA) and culture with Eagle’s Minimum Essential Medium containing 2 mM L-glutamine, 1 mM sodium pyruvate, Minimum Essential Medium (MEM) non-essential amino acid, heat-inactivated 10% fetal bovine serum (FBS), 100 µg/mL streptomycin and 100 IU/mL penicillin (all compounds from Sigma-Aldrich, Darmstadt, Germany). Cultures were maintained at 37 °C in a 95% humidified atmosphere of 5% CO_2_. The culture medium was changed every 2–3 days. The cells were passaged using 0.25% trypsin- ethylenediaminetetraacetic acid (EDTA) solution (Sigma-Aldrich, Darmstadt, Germany) when reaching 70–80% of confluence.

Murine fibroblast cell line L929 (American Type Culture Collection, Manassas, VA, USA) were cultured at 37 °C in 5% CO_2_ and 95% humidity in a complete Roswell Park Memorial Institute (RPMI) 1640 medium containing 2 mM L-glutamine, heat-inactivated 10% FBS, 100 µg/mL streptomycin and 100 IU/mL penicillin. L929 cells culture conditions were the same as we have described previously [32].

The cells belong to both cell lines, in a volume of 1 mL of appropriate culture medium, were seeded onto the autoclaved tested specimens placed in a 24-well culture plate (Corning, Corning, NY, USA) at a density of 1 × 10^4^ cells/well for 24 h, 72 h or 120 h, respectively. The cells incubated with the tested plates in the above incubation time were also analyzed for the observation of cell morphology. Cell morphology and proliferation was investigated for the Ti6Al4V/TNH samples as well as for Ti6Al4V/TNT specimens.

#### 2.3.2. Proliferation of L929 Fibroblasts and MG-63 Osteoblasts Detected by MTT Assay

The effect of the tested specimens on the cell proliferation (after 24-, 72- and 120 h, respectively) was studied by the MTT (3-(4,5-dimethylthiazole-2-yl)-2,5-diphenyl tetrazolium bromide; Sigma Aldrich, Darmstadt, Germany) assay using the same method as it was reported in Lewandowska et al. [28]. Briefly, after the respective incubation time, the plates were washed with phosphate buffered saline (PBS) and transferred to a new 24-well culture plate. The MTT (5 mg/mL; Sigma-Aldrich, Darmstadt, Germany) solution in a culture medium without phenol red (RPMI 1640 medium for L929 fibroblasts or Eagle’s Minimum Essential Medium for MG-63 osteoblasts; both from Sigma-Aldrich, Darmstadt, Germany) was added to each well. After 3 h of incubation at 37 °C in a humidified atmosphere of 5% CO_2_, the solution was aspirated, 500 μL of dimethyl sulfoxide (DMSO; 100% *v/v*; Sigma Aldrich, Darmstadt, Germany) was added to each well and the plates were shaken for 10 min. The absorbance was measured at the wavelength of 570 nm with the subtraction of the 630 nm background, using a microplate reader (Synergy HT; BioTek, Winooski, VT, USA). The blank groups (the plates incubated without cells) were treated with the same procedures as the experimental groups. All measurements were done in duplicate in five independent experiments.

#### 2.3.3. Cell Morphology

Scanning electron microscopy (SEM; Quanta 3D FEG; Carl Zeiss, Göttingen, Germany) analyses were performed to study the morphology changes of L929 fibroblasts and MG-63 osteoblasts grown on the surface of the tested specimens using the same method as in Lewandowska et al. [32]. Briefly, after the selected incubation time, the samples were washed with PBS to remove the non-adherent cells and were fixed in 2.5% *v/v* glutaraldehyde (Sigma Aldrich, Darmstadt, Germany) for a minimum of 4 h. Then, the plates were rinsed again with PBS and dehydrated in a graded series of ethanol (50%, 75%, 90%, and 100%). Finally, the specimens were dried in vacuum-assisted desiccators overnight and stored at room temperature until the SEM analysis was performed.

#### 2.3.4. Statistical Analysis in the MTT Assay

All values are reported as means ± standard error of the means (SEM) and were analyzed using analysis of variance (ANOVA) followed by Bonferroni multiple comparisons test with the level of significance set at *p* < 0.05. Statistical analyses were performed with GraphPad Prism 7.0 (GraphPad Software, La Jolla, CA, USA).

## 3. Results

### 3.1. Synthesis of Nanotubular Coatings and Analysis of Their Surface Morphology on Different Steps of Experimental Procedure

**Figure 1:**
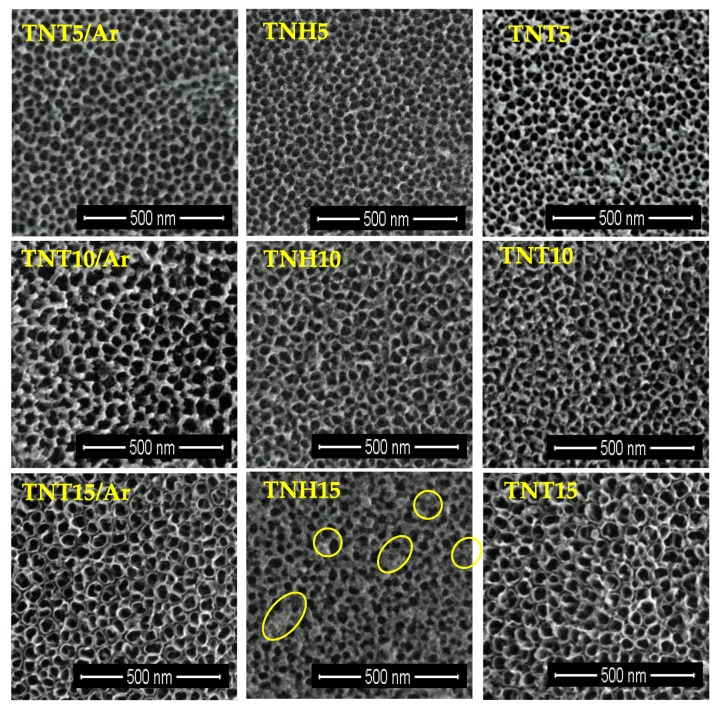
Scanning Electron Microscopy (SEM) images of Ti6Al4V/TNT5-15/Ar, Ti6Al4V/TNH5-TNH15, and Ti6Al4V/TNT5-15 samples surface (selected destruction sites of TiO_2_ nanotubes were marked with circles).

**Figure 2:**
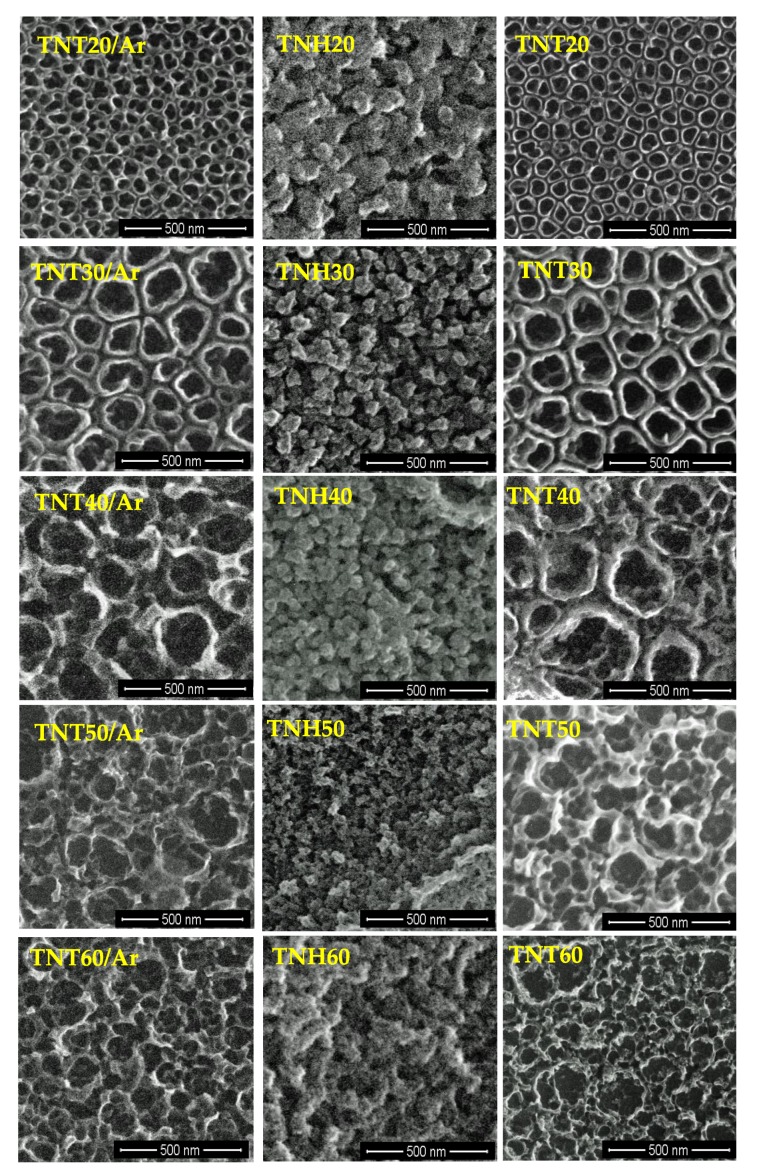
SEM images of Ti6Al4V/TNT20-60/Ar, Ti6Al4V/TNH20-TNH60, and Ti6Al4V/TNT20-60 samples surface.

Titania nanotube (TNT) layers on the surface of Ti6Al4V foil samples were produced according to the earlier described electrochemical oxidation methodology (anodization process) [25] in potentials range *U* = 5–60 V at room temperature (RT). After washing them in deionized water and drying them in the stream of argon their surface morphology has been checked. SEM images presented Ti6Al4V/TNT/Ar surface nanoarchitecture are visible in Figure 1 and Figure 2. In Table S1 diameters of formed nanotubes and their wall thickness are presented.

In the next stage, the produced Ti6Al4V/TNT5-TNT60/Ar systems were divided into two groups. The first one was autoclaved directly, leading to the production of Ti6Al4V/TNH5-TNH60, and the second one, before autoclaving was subjected to the additional drying process, using immersion in acetone and drying at 396 K for 1 h. This second procedure led to the production of Ti6Al4V/TNT5-TNT60. Figure 1 and Figure 2 present the differences in surface morphology of Ti6Al4V/TNH5-TNH60 and Ti6Al4V/TNT5-TNT60 and their comparison with the initial nanotubes samples, after drying in Ar stream, before the autoclaving process - Ti6Al4V/TNT5-TNT60/Ar. The comparison of SEM images of TNT5/Ar, TNT10/Ar coatings with TNH5, TNH10, TNT5, and TNT10 ones proves that regardless of the method used to dry the samples before autoclaving, the surface morphology after autoclaving remains unchanged, even identical (Figure 1). In the case of TNT15/Ar, TNH15, and TNT15 coatings, the slight surface changes were noticed for the TNH15 sample, which consisted in the partial, incidentally appeared destruction of nanotubes. Analysis of SEM images of TNT20-TNT60 coatings and TNH20-TNH60 ones revealed significant differences in their morphology. The tubular surface architecture of Ti6Al4V/TNT20-60 is identical with the initial samples Ti6Al4V/TNT20-60/Ar. But the surface architecture of Ti6Al4V/TNH20-60 samples is nothing like the starting nanotubes—nanotubular architecture of Ti6Al4V/TNT20-60/Ar was completely destroyed (Figure 2). 

A significant influence of preparing procedure of nanotubular coatings for autoclaving and the autoclaving process itself, on surface morphology changes, for nanotubes with higher potential (20–60 V), highlighted a focus for future works in terms of structure, wettability, mechanical properties, and biocompability of two groups of samples obtained at potential 20–60 V: (1) nanotubes coatings, dried after their production in ordinary way, only with Ar stream at RT—which nanotubular morphology during the autoclaving is completely destroyed (we will describe them as Ti6Al4V/TNH20-60 systems, or just TNH20-60 coatings, H—indicates that they are hydrothermally modified) and (2) nanotubes coatings dried additionally using immersion in acetone and slow drying in 396K, which nanotubular morphology during the autoclaving is not changed (we will describe them as Ti6Al4V/TNT20-60 systems or just TNT20-60 coatings).

### 3.2. Structural Studies on TNH20-60 and TNT20-60 Coatings and Their *Wettability Analysis*

**Figure 3:**
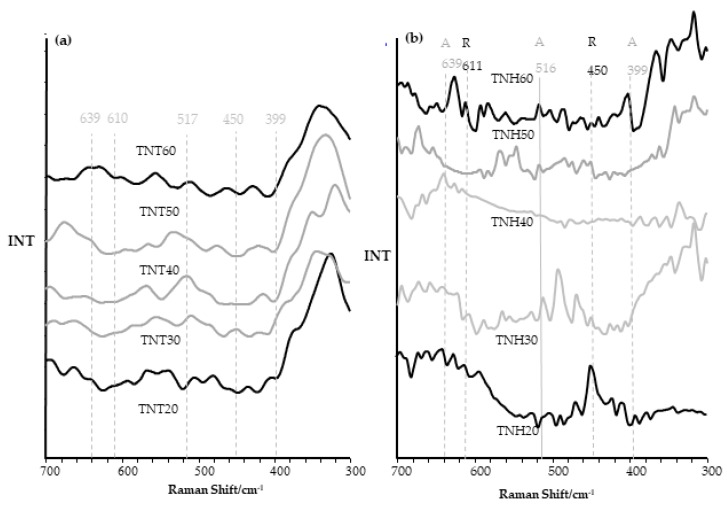
Raman spectra of Ti6Al4V/TNT20-60 (**a**) and Ti6Al4V/TNH20-TNT60 (**b**) samples (A—TiO_2_ anatase form, R—TiO_2_ rutile form).

The Raman and diffuse reflectance infrared Fourier transform spectroscopy (IR DRIFT) methods have been used to study the eventual structural differences between Ti6Al4V/TNH20-TNT60 and Ti6Al4V/TNT20-60 systems, as we suspected that structural changes could follow the already described morphological changes (Figure 3). Analysis of Raman spectra between 300 and 700 cm^−1^ of TNT20-TNT60 coatings confirms the amorphousness of these samples (Figure 3a). Raman spectra of TNH20-TNH60 samples indicate also on the formation of amorphous layers. However, very weak bands, which were found at 450 and 611 cm^−1^ and also at 399, 516, and 639 cm^−1^ indicate on the possible phase transitions and the formation of TiO_2_ rutile/anatase (TNH20, TNH30, TNH60) nanocrystals (Figure 3b). The strong bands detected between 600 and 950 cm^−1^ in all DRIFT spectra of TNH20-TNH60 samples confirm the formation of TiO_2_ layers (Figure S1). 

**Figure 4:**
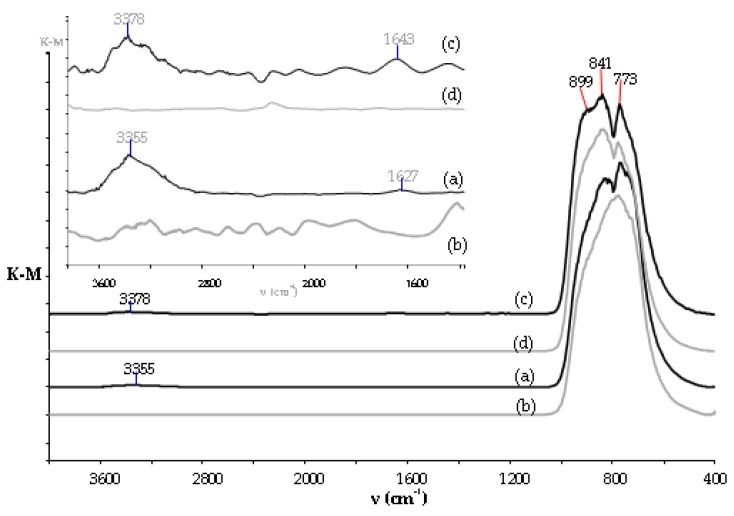
Infrared (IR) spectra (DRIFT) of (**a**) Ti6Al4V/TNT20/Ar and (**c**) Ti6Al4V/TNT50/Ar samples (the samples after drying in the Ar stream) and Ti6Al4V/TNT20/Ac (**b**) and Ti6Al4V/TNT50/Ac (**d**) the samples immersed in acetone and dried at 396 K by 1 h.

**Figure 5:**
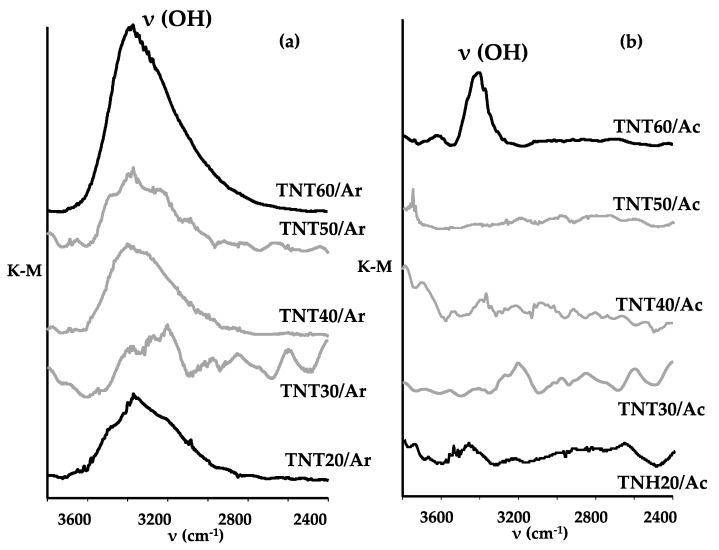
IR DRIFT spectra of (**a**) Ti6Al4V/TNT20-60/Ar and (**b**) Ti6Al4V/TNT20-60/Ac systems.

In order to answer the question, what is responsible for the differences in the surface morphology of Ti6Al4V/TNT20-60 and Ti6Al4V/TNH20-TNT60, and at the same time taking into account our suspicion that the reason for the differences may be the water, which is not completely dried during the traditional drying process using a stream of argon, we made detailed Raman and IR DRIFT spectra analyses of Ti6Al4V/TNT20-60/Ar and Ti6Al4V/TNT20-60/Ac systems. Analysis of DRIFT spectra of Ti6Al4V/TNT20-60/Ar samples revealed the presence of weak and very weak bands at 3320–3390 cm^−1^ and 1620–1660 cm^−1^, which were attributed to ν(OH) (stretching) and δ(HOH) (bending) modes of water molecules, respectively (Figure 4 and Figure 5). Moreover, the very strong band, which was found between 450 and 1000 cm^−1^ was assigned to ν(Ti-O) modes of TiO_2_, which confirms the formation of titania nanotube layers. In IR spectra of Ti6Al4V/TNT20-60/Ac samples (which after anodization were immersed in acetone and dried at 396 K), the intensity of bands attributed to vibrations of water molecules significantly decreased (Figure 5). According to these data, we can assume that the use of additional drying procedure allows for the removing of water molecules from the nanotubular surface of Ti6Al4V/TNT20-60/Ar, in particular from inside the nanotubes.

The results of contact angles measurements for water and diiodomethane, and also changes of surface free energy value (SFE) of Ti6Al4V/TNH20-60 and Ti6Al4V/TNT20-60 are presented in Figure S2 and in Table S2. According to these data, it can be stated that the wettability of Ti6Al4V/TNH20-60 layers is significantly different then adequate values for Ti6Al4V/TNT20-60. However, these differences in the case of TNT60 and TNH60 are not so huge. Analysis of data presented in Figure S2(a) indicate the clear hydrophobic character of Ti6Al4V/TNH20-60 layers, whose tubular architecture was destroyed and much more hydrophilic character of Ti6Al4V/TNT20-60. In the case of Ti6Al4V/TNT20-60 their hydrophilicity decreases from TNT20 to TNT60. The free surface energy (SFE) of the produced coatings was appointed by the Owens-Wendt method [33]. This method required the contact angles measured for two liquids, i.e., water as a polar liquid (Figure S2(a)) and dispersive one such as diiodomethane (Figure S2(b)). The SFE calculations for Ti6Al4V/TNT20-60 samples showed that their values change in the narrow range, i.e. from SFE = 47.6 (mJ/cm^2^) up to SFE = 53.7 (mJ/cm^2^). In the case of Ti6Al4V/TNH20-60 samples, the SFE value increases from 28.4 to 63.8 (mJ/cm^2^) for TNH20-TNH40 and again decreases to 61.0 and 49.2 (mJ/cm^2^) for TNH50-TNH60 respectively (Figure S2(c)).

### 3.3. Topography and Mechanical Properties of Ti6Al4V/TNH20-60 and Ti6Al4V/TNT20-60 Samples

The studies of surface topography and mechanical properties (such us hardness, Young’s modulus) were carried out on the reference Ti6Al4V specimens, Ti6Al4V/TNH20-60 and Ti6Al4V/TNT20-60 systems. The studies of adhesion were performed for the same composites without reference Ti6Al4V specimens. The purpose of the research was to determine the relations between roughness parameters S_a_, of studied systems.

#### 3.3.1. Surface Topography 

**Figure 6:**
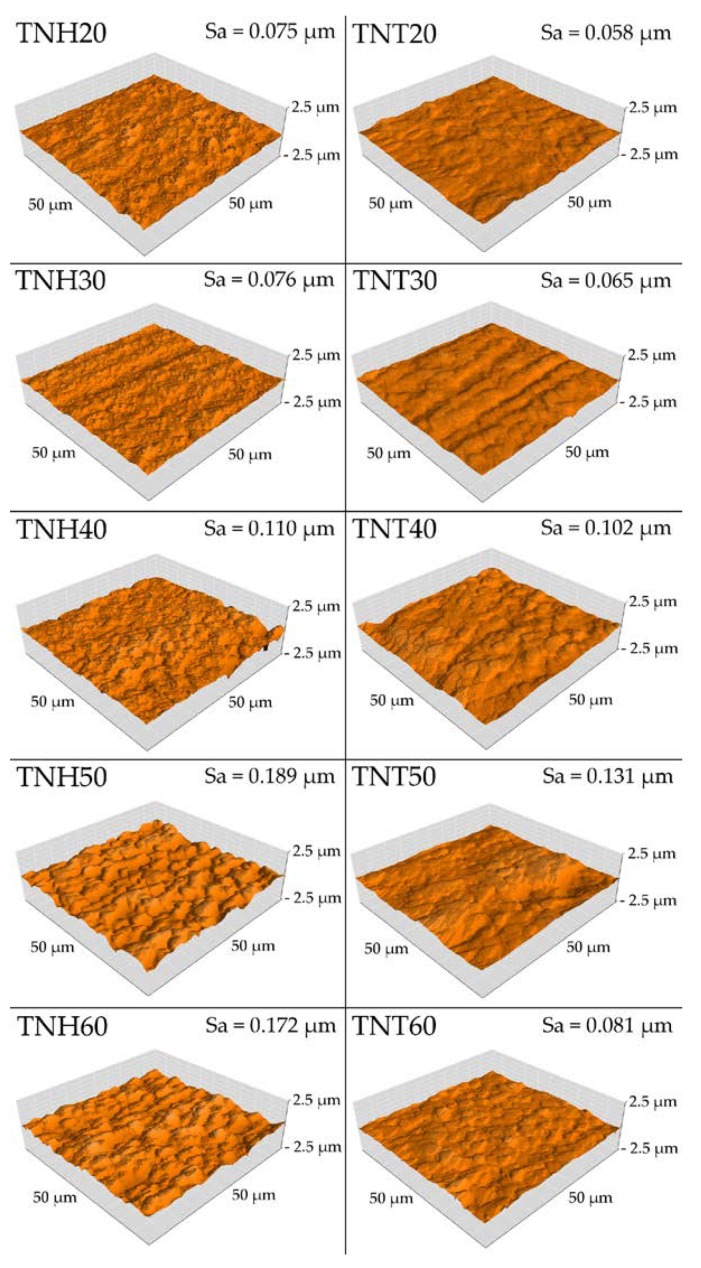
Atomic force microscopy (AFM) topography of Ti6Al4V/TNH20-60 and Ti6Al4V/TNT20-60 systems.

Analysis of atomic force microscopy (AFM) images allowed the estimation of differences in surface topography of Ti6Al4V/TNH20-60 and Ti6Al4V/TNT20-60 samples versus pure Ti6Al4V foil as a reference sample. Surface roughness parameter S_a_, was determined using software, being an integral part of the device. The AFM topography and S_a_ parameters value are presented in Figure 6 and in Table S3. As demonstrated by the performed research, the electrochemical anodization of titanium alloy surface and their further autoclaving increases the roughness parameter S_a_ for all specimens in comparison to the reference titanium alloy. For the layers from TNT20 to TNT50 and TNH20 to TNH50, the increase of roughness with increasing voltage applied during the anodization process was observed. For the TNT60 and TNH60 layers, the decrease of the roughness was noticed compared to earlier TNT50 and TNH50 coatings. Also, the additional drying of Ti6Al4V/TNT/Ar changed surface roughness parameter S_a_ of Ti6Al4V/TNH20-60—all of them posses the higher S_a_ values than adequate Ti6Al4V/TNT20-60 systems. The correlation between the S_a_ parameter and the architecture of the nanotubular layers can be observed (Table 1, Table S1, Figure 2, Figure 6). With increasing the wall thickness of nanotubes, the roughness parameter S_a_ increased.

#### 3.3.2. Mechanical Properties (Hardness and Young’s Modulus) of Ti6Al4V/TNH20-60 and Ti6Al4V/TNT20-60 Systems

The nanomechanical properties of reference Ti6Al4V specimen, Ti6Al4V/TNH20-60 and Ti6Al4V/TNT20-60 systems, such as hardness and Young’s modulus are presented in Table 1. Nanoindentation technique, which is dedicated to mechanical studies of nanometric structures, was used to obtain the results. The anodization of Ti6Al4V samples with the potential 20–60 V and their further treatment (drying in Ar and autoclaving or drying in Ar, immersion in acetone, additional drying and autoclaving) completely changed mechanical properties all of the tested specimens but in a different way for different samples. Only for Ti6Al4V/TNT20 and Ti6Al4V/TNT30 the increase of mechanical properties as compared to the reference material was observed. For TNT40 and TNT50, which among Ti6Al4V/TNT20-60 systems was characterised by the greatest diameter of nanotubes, the deepest decrease in hardness and Young’s modulus were demonstrated. In the case of Ti6Al4V/TNH20-TNH60 samples, all tested specimens revealed lower mechanical properties than the reference titanium alloy and about 50% lower hardness than the same specimen additionally dried before hydrothermal treatment—Ti6Al4V/TNT20-60. The remarkable standard deviations for mechanical properties are characteristic for the nanoindentation technique and confirm the accuracy of measurements. 

#### 3.3.3. Adhesion Properties

In Table 2 the results of adhesion tests of the Ti6Al4V/TNT20-60 and Ti6Al4V/TNH20-60 systems are presented. 

The critical load was assumed as the maximum force at which the nanotubular or nanostructural layers were delaminated from titanium alloy substrate, and critical friction as the maximum friction force during layers` delamination. Scratch-tests showed lower values of critical load and critical friction for Ti6Al4V/TNH20-60 systems comparing to Ti6Al4V/TNT20-60 ones. It means that the adhesion of non-tubular coatings is lower than adequate tubular ones. And that additional drying of nanotubular samples before autoclaving increases the adhesion to titanium alloy substrate. The biggest difference in the critical force (delamination force) between Ti6Al4V/TNT20-60 and Ti6Al4V/TNH20-60 systems is visible for samples TNT30 and TNH30 and is equal 25%, while the smallest difference is visible for TNT60 and TNH60 (12%).

### 3.4. Cell Proliferation Detected by MTT Assay

**Figure 7:**
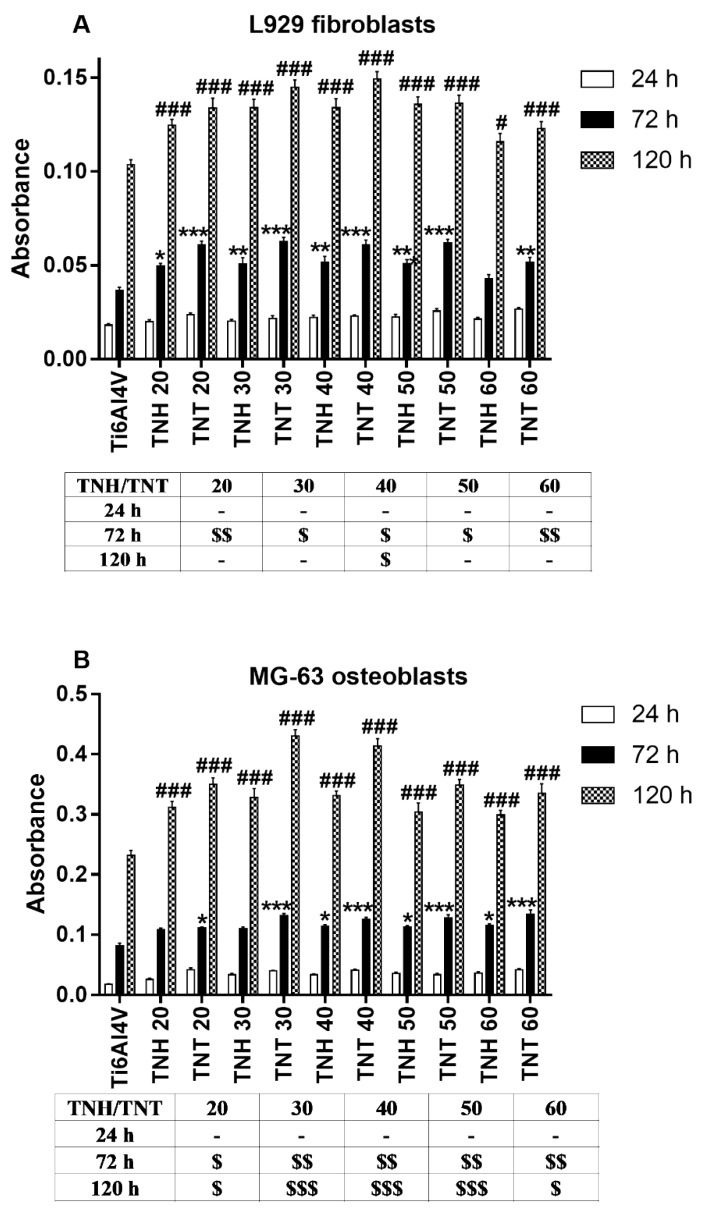
L929 murine fibroblasts (**A**) and human osteoblasts MG-63 (**B**) proliferation (after 24-, 72- and 120 h) on the surface of Ti6Al4V/TNH20-TNH60 and Ti6Al4V/TNT20-TNT60 samples, detected by MTT assay. The absorbance values are expressed as means ± SEM of five independent experiments. Asterisk and hash mark indicate significant differences between the cells incubated with the alloy references samples (Ti6Al4V) compared to the TNH and TNT specimens after 72 h (* *p* < 0.05, ** *p* < 0.01, *** *p* < 0.001) or 120 h (^#^ *p* < 0.05, ^##^ *p* < 0.01, ^###^ *p* < 0.001) of incubation time, respectively. Tables below the graphs A and B present the statistical differences in the proliferation of the cells, between TNH and TNT coatings produced by the electrochemical anodization of the Ti6Al4V foil at the same selected potential (^$^ *p* < 0.05, ^$$^ *p* < 0.01, ^$$$^ *p* < 0.001).

Biointegration of Ti6Al4V/TNH20-60 and Ti6Al4V/TNT20-60 systems were evaluated based on the results of the MTT (3-(4,5-dimethylthiazol-2-yl)-2,5- diphenyltetrazolium bromide) assays made for the two different cell lines: murine L929 fibroblasts and human osteoblast-like MG 63 cells. The level of proliferation (assessed after 24-, 72- and 120 h) of the cells growing on the Ti6Al4V/TNH20-60 surface was compared to that observed for the cells cultured on the Ti6Al4V/TNT20-60 e.g., TNH20 *vs.* TNT20. As it can be seen in Figure 7, with an increase of incubation time more L929 fibroblasts (Figure 7A), as well as MG-63 osteoblasts (Figure 7B), proliferated on the surface of the all tested biomaterials (*p* < 0.001). 

Analysis of these data revealed also that there were no differences in the cells proliferation after 24 h between the all tested samples and titanium alloy references sample (Ti6Al4V). In contrast, Ti6Al4V/TNH coatings, as well as Ti6Al4V/TNT nanolayers, provoked a significant increase in cells proliferation compared to the Ti6Al4V reference alloy. This phenomenon was the most noticeable after 120 h of incubation both in the culture of fibroblasts (with the exception of TNH60 sample) and osteoblasts (*p* < 0.001). In the tables below the graphs in Figure 7, the statistical differences in the cells biointegration between TNH and TNT coatings produced by the electrochemical anodization of the Ti6Al4V foil at the same selected potential were presented. Analysis of these tables shows that there were no differences in cells proliferation measured after 24 h for both tested cells lines. In contrast, TNT nanolayers caused a greater increase in MG-63 osteoblasts proliferation than TNH coatings, which was noticed after 72 h as well as 120 h of incubation time (Table in Figure 7B). On the other hand, L929 fibroblasts cultured on the surface of TNT samples showed a higher rate of proliferation than the cells that grew on the TNH specimens only after 72 h of incubation time, except for the comparison of TNH40 and TNT40 layers, when the differences were also observed after 120 h (Table in Figure 7A). However, it should be clearly emphasized that all the tested TNH coatings showed a much higher level of biocompatibility than titanium alloy references sample (Ti6Al4V) and TNT coatings even higher than TNH ones.

### 3.5. Cell Morphology Observed by Scanning Electron Microscopy

**Figure 8:**
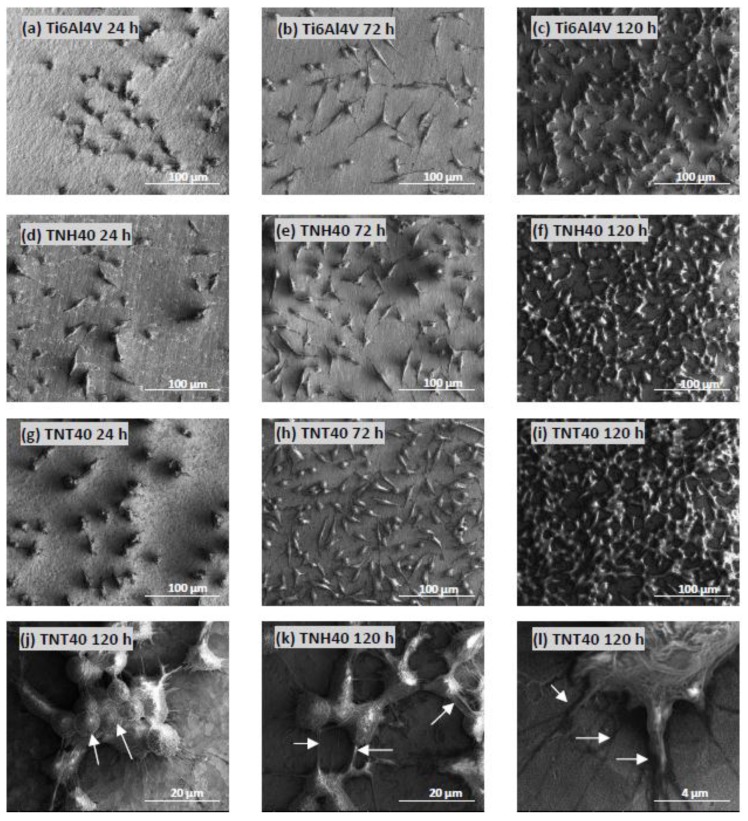
Scanning electron microscopy (SEM) images presenting proliferation of the murine L929 fibroblasts growing on the surface of the titanium alloy references sample (Ti6Al4V; (**a**–**c**) in comparison with Ti6Al4V/TNH40 sample; (**d**–**f**) and Ti6Al4V/TNT40 sample; (**g**–**i**). Arrows in the image (**j**) show the cells growing in layers on top of each other. Arrows in micrographs (**k**) and (**l**) indicate filopodia spread between cells or penetrating deep into the TNT40 nanolayers, respectively

**Figure 9:**
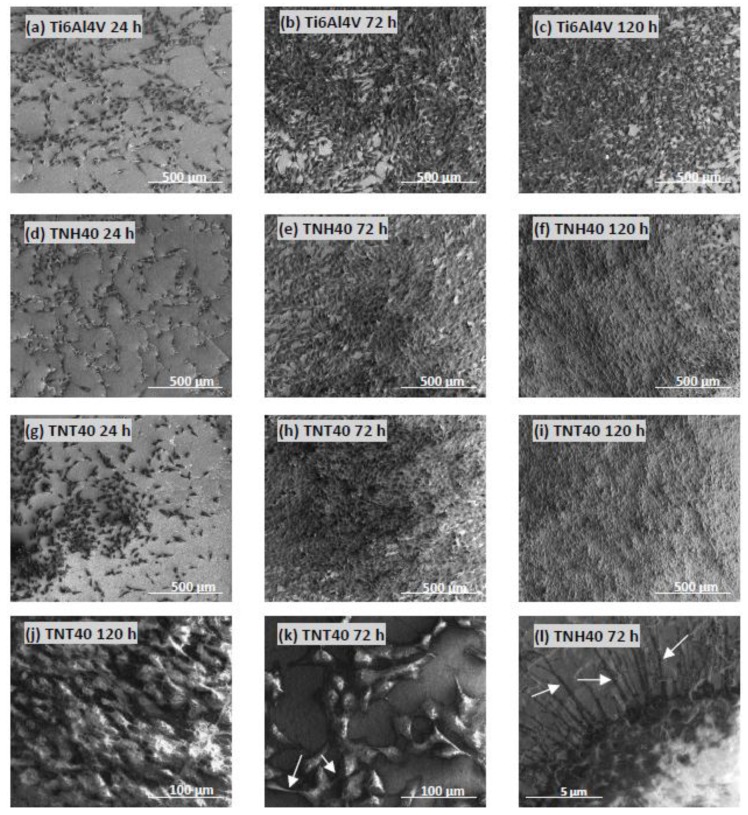
Scanning electron microscopy (SEM) micrographs showing the human osteoblast-like MG-63 cell proliferation on the surface of the references sample (Ti6Al4V; (**a**–**c**) compared to Ti6Al4V/TNH40 sample; (**d**–**f**) and the Ti6Al4V/TNT40 sample; (**g**–**i**). Micrograph (**j**) presents the multilayer growth of cells. Arrows indicate numerous filopodia spreading between cells (**k**) or filopodia, which attached osteoblasts to the nanocoatings surface (**l**).

SEM images micrographs present L929 murine fibroblasts (Figure 8) and MG-63 human osteoblasts (Figure 9) morphology, and proliferation. The cells showed of both figures were cultured on the surface of Ti6Al4V references sample, Ti6Al4V/TNH40, and Ti6Al4V/TNT40 nanocomposites. These data support the MTT results and clearly demonstrate the high biocompatibility properties of both types of tested nanomaterials, which are mainly related to the increase in cells proliferation level over time (compare micrographs (a–c), (d–f) and (g–i) in Figure 8 and Figure 9, respectively). 

Importantly, as it can be seen in Figure 8j, L929 fibroblasts also start to grow in layers on top of each other and this phenomenon is much more noticeable during the MG-63 osteoblasts incubation (see, e.g., Figure 9j), when the entire surface of TNH40 as well as TNT40 coatings is overgrown with multilayer structure of growing cells after 120 h of incubation time (Figure 9f,i, respectively). Finally, SEM images show that L929 fibroblasts as well as MG-63 osteoblasts form numerous filopodia, which strongly attach the cells to the nanocoatings surface by penetrating deep into nanolayers (arrows in Figure 8l and Figure 9l, respectively) or the cells generate filopodia between themselves (arrows in Figure 8k and Figure 9k, respectively).

## 4. Discussion

During designing and manufacturing titanium alloy implants, we focused mainly on ensuring their surface the appropriate physicochemical and mechanical properties, which directly influence on implants biointegration and anti-inflammation activity [34,35]. Providing the basic aseptic and sterile properties to implants is another important issue requiring a solution at the design level. This is particularly important when their surface is modified by a nanocomposite coating. The understanding of how the sterilization process influences on the implant surface properties may be important for the future clinical outcome. The most common method of sterilization is the steam sterilization (autoclaving), during which irreversible damage of microorganisms takes place due to the hydrothermal processes taking place. In our works, we have focused on autoclaving influence on surface layer morphology, structure, wettability, mechanical properties, and bioactivity of Ti6Al4V/TNT*U* systems produced by anodic oxidation using potentials *U* = 20–60 V. Spectral studies (DRIFT) of TNT coatings after anodization (dried only Ar stream at room temperature) proved the presence of traces of water on their surface (Figure 4). It can be a result of the adsorption of water molecules on the titania layer surface coming from washing of samples in deionized water after anodization. In other cases, the use of additional drying procedures to remove adsorbed water from the TNT layers surface is necessary. The good results were obtained by the immersion of samples in acetone (10 min., ultrasonic bath) and then their drying at 396 K for 1h. DRIFT spectra presented in Figure 3 and Figure 4 confirmed the decrease of intensity of bands assigned to water molecules. 

Analysis of SEM images of TNT5-15 and TNH5-15 coatings exhibited the lack of significant differences between surface morphology of samples produced at the same conditions but subjected to different drying procedure. It suggests that the surface morphology of TNT layers composed of densely packed nanotubes of diameter 25–70 nm, does not change as a result their interaction with hot water vapor (396 K) under high pressure (120 kPa) (Figure 1). The results of our earlier studies of Ti6Al4V/TNT*U* composites (TNT*U* layers were produced using electrochemical oxidation at potentials *U* = 3–20 V) revealed that coatings, which consisted of densely packed tubes of diameter c.a. 20 nm (TNT4-TNT8) exhibited the hydrophobic character, which decreased with the increase of their diameter and separation (TNT10-TNT20) [25]. Analysis of these data suggests that the hydrophobic character of TNT coatings produced at lower potentials (*U* = 5–10 V), causes that the use of Ar stream to remove of adsorbed water traces should be sufficient. Although, on the surface of TNH15 sample the slight changes, which were the result of the destruction of small tubular architecture fragments, have been found (Figure 1). The autoclaving of Ti6Al4V/TNT/Ar systems led to the complete destruction of their tubular architecture, while the surface morphology of Ti6Al4V/TNT/Ar samples remained unchanged (Figure 2). The affecting of the TNT layers drying on the stabilization of their tubular architecture was also proved by the results of our previous works on the use of TNT coatings, as the substrates in chemical vapor deposition (CVD) of silver nanoparticles [36,37]. According to these reports, the amorphous TNT coatings kept their tubular architecture during their heating up to 693 K in the stream of dry argon (carrier gas). Simultaneously, the autoclaving of all produced materials after CVD experiments did not change their surface morphology and amorphousness. The coatings consisted of anatase or anatase/rutile nanocrystals were formed during heating of the TNT coatings above 693 K [32]. The results of Liu et al investigations revealed that the annealing of amorphous TNT layers at 723 K for 1h led to the phase transitions up to TiO_2_ anatase form (thermally annealed and “water annealed” samples) or the anatase/rutile mixture (hydrothermally treated sample) [38]. According to this report, the hydrothermal treatment led to faceting or granularization of the tube walls, while the use of both other methods mentioned above, led to destroying their tubular architecture. The destruction effect of autoclaving carried out on amorphous TNT layers (tubes of diameters 15 and 50 nm), was also noticed by Junkar et al. [39]. In this case, the observed surface morphology changes have been explained as a combined result of the effect of moisture and temperature c.a. 365 K, which caused the crystallization of amorphous TNT layers. The results of Lamberti et al. studies also revealed that amorphous titania nanotubes crystallized into TiO_2_ anatase form after exposure to water vapor in ambient conditions [40]. The transformation of TNT layers to anatase or anatase/brukite phase, treated by the hot deionized water (365 K) for 35h, were noticed by Liao et al [41]. Studies of Liu J. et al. showed that the nanotubular architecture of the amorphous titania nanotubes remained almost unchanged during the hydrothermal reaction at 403–453K for 1h [42]. The prolongation of the hydrothermal reaction time to 4, 6h caused that TiO_2_ nanotubes were converted into nanoparticulate aggregations, which narrow the tubes’ diameters up to their closure. Considering previous reports, it should be noted that that phase transitions of amorphous titania nanotubes to polycrystalline TiO_2_ forms and surface morphology changes TNT layers can result in the water molecules interactions with the nanotube walls, which probable catalyzes the rearrangement of TiO_6_^2−^ octahedra and initiate crystallization processes [39,40,42]. 

Discussing the observed changes of TNH20-TNH60 coatings morphology after their autoclaving, it should be pointed out the possible influence of water molecules adsorbed on/inside the TNT coating surface. The adsorbed water molecules on the TNT surface in contact with the hot steam under the higher pressure accelerates the tubular architecture destroying and simultaneously favors the phase transition of the amorphous nanotubes to TiO_2_ polycrystalline forms. Analysis of Raman spectra of TNH20-TNH60 layers revealed the appearance of very weak bands at 450 and 611 cm^−1^ and at 399, 516, and 639 cm^−1^, which would indicate on the formation of rutile or rutile/anatase nanocrystals (Figure 5).

The comparison of TNH20-TNH60 and TNT20-TNT60 wettability data revealed a significant hydrophobic character of TNH20-TNH50 coatings, although the trend of changes is not strictly defined. The contact angle increases in the row: TNH20<TNH30<TNH40 and decreases in the row: TNH40>TNH50>TNH60, pointing out the sample TNH40 as the most hydrophobic. SEM analysis of this sample surface showed that coating is characterized by very dense packing, which does not allow the penetration of water molecules when measuring the contact angle. In comparison, TNT coatings are more hydrophilic, however their hydrophilicity decreases with the increase of diameter of tubes, and the lowest contact angle value is assigned to TNT20. What should be underlined the contact angle of TNH60 and TNT60 is almost identical, but this similarity should not come as a surprise - if you look at SEM images of both systems, you can also see some similarity in the morphology of the surface (Figure 2 and Figure S2).

The use of AFM technique permitted to characterize the topography and, dedicated for studied coatings and layers, S_a_ roughness parameters. It was proven than roughness of the implant’s surface would indeed be an important factor determining the proper osseointegration process between implants and bone. The high value of the surface roughness brought out better osteoblast adhesion after implementation in primary stabilization phase [43]. In this study, the positive effect of anodization of titanium alloy resulting in higher surface roughness was proved as compared with reference Ti6Al4V and additionally confirmed by biological tests. The same effect was reported previously [44,45]. Analysis of AFM images of TNH20-60 and TNT20-60 proved the influence of the presence of trace of water on/inside nanotubes and further autoclaving on the surface topography. The S_a_ parameters are higher for TNH20-60 coatings than for TNT20-60 ones, pointing out that disappearance of nanotubular architecture and the creation of granular nanoarchitecture leads to more porous surface and gives higher values of S_a_ (Figure 2 and Figure 6).

The mechanical properties of all studied specimens were measured by nanoindentation technique. Noticeable standard deviations were obtained, which can be mostly attributed to the morphology of TiO_2_ layers (such as presence of pores and nanotubular architecture). For Ti6Al4V specimen hardness was equal to 16.17 ± 3.61 GPa, and only TNT20 and TNT30 specimens demonstrated higher values of hardness. The mechanical properties, such as hardness and Young’s modulus, and adhesion strength of layers for long-term and load-bearing implants determine their applicability for the living organisms. It has been proven that high difference between mechanical properties of bone and implants can increase a danger of a so-called “shielding effect” and in consequence of destabilization and loosening of the implants [46,47]]. Mechanical properties of nanotubular layers obtained by nanoindentation technique were dependent on the morphology of TiO_2_. It was observed that increasing hardness followed indenter penetration depth increase. The influence of microstructural features such as columns’ type, crystalline or amorphous character of the layer and porosity on mechanical properties of TiO_2_ has been proven [48]. The tubular’ architecture of the layers on titanium alloy determine mechanical properties. The nanotube walls are characterized by very high mechanical properties, such as hardness. The decrease of measured properties of the layers TNH20-60, compared with TNT20-60, can be explained by homogenization of the surface and the disappearance of nanotubular architecture on all of the specimens TNH20-60. The increase in packing density and porosity of TiO_2_ were already reported to cause an increase of hardness and Young`s modulus value [49]. According to SEM images (Figure 2) of specimens with visible nanotubular architecture (TNT20-60), the porosity and packing density are the highest for TNT20 and TNT30 specimens, which possess the highest mechanical properties. The highest value of hardness of TNH20 among all TNH specimens may be due to confirmed presence of rutile in the structure of TNH20 tubular layer, the highest among all TNH20-60 specimens [50]. The ratio of H (hardness) to E (Young’s modulus) H^2^/E^3^ describes the resistance of the material to plastic deformation [51]. The highest value of H^2^/E^3^ was noticed for Ti6Al4V specimens (0.058 GPa), which suggests better tribological properties of reference specimen than TNT and TNH layers [52]. Nanoscratch-test was used in the past to study adhesion of thin oxide layer on titanium alloys [53,54]. Adhesion between the layer and the implant surface is one of the most important factors deciding on the possibility of using this modification technique for the biomaterials. Loosening of layer crystals to the tissue surrounding the implant can cause inflammatory states. In this study, TNT20-60 layers, which have higher mechanical properties, were characterized by better adhesion to the titanium substrate. TNH20-60 samples are characterized by decreased the critical force (delamination force) due to decreased hardness of the layers. Furthermore, decrease of adhesion of the layers was caused by change in the roughness of the surface. For all tested TNH specimens, the roughness was higher than for adequate TNT coatings. Cedillo-Gonzalez et al. [55] proved that higher roughness of the layers reduced adhesion.

In the present study, the biointegration of tested nanomaterials was investigated using two cell lines: mouse L929 fibroblasts and human osteoblast-like MG-63 cells. Since it is well known that the long-term success of an implant depends not only on the integrity of osseointegration but also on the contact with surrounding soft tissue [56], the use of both osteoblasts and fibroblasts cell lines in *in vitro* studies allowed for a comprehensive examination of implant biocompatibility. Moreover, it is well established that cellular behavior, such as adhesion, morphologic change, including formation of filopodia, and proliferation is determined by surface properties of nanomaterials, thus cellular response measured by these parameters are required to assess the biointegration of implants [57]. In our study, this response was estimated using MTT assay and scanning electron microscopy images analysis. The results of MTT assays related to the cell proliferation (measured after 24- 72- and 120 h) revealed the promising biocompatible properties both of TNT20-TNT60 as well as TNH20-TNH60 samples, which were observed both during fibroblast as well as osteoblasts culture (Figure 7A,B, respectively). Moreover, the analysis of these data revealed a slight deterioration of proliferation properties of TNH20-TNH60 samples in comparison to TNT20-TNT60 ones, both for fibroblast as well as osteoblasts culture. These findings were confirmed by SEM micrographs analysis that unambiguously indicate the increase in the number of cells over time that was greater for TNT40 and TNH40 surface compared to the Ti6Al4V references sample (Figure 8 and Figure 9, respectively). Importantly, the cells, especially MG-63 osteoblasts, have overgrown entire surface of TNT40 and TNH40 nanocoatings forming multilayer structure of growing cells (Figure 9j). Since it is believed that the stimulation of post-confluent osteoblasts proliferation result in the formation multilayered nodules that later become mineralize [58], we presume that the tested specimens can stimulate bone regeneration *in vivo* due to the high osseointegration level. Finally, the biocompatibility of tested nanomaterials was also confirmed by the filopodia’s formation between the cells (arrows in Figure 8k and Figure 9k, respectively) growing on the tested nanomaterials, which attached also the cells to the coating’s surface (arrows in Figure 8l and Figure 9l, respectively) by penetration inside the porous nanolayer. Consequently, the porous surface caused a stronger cell adhesion by functioning as anchorage points for fibroblasts as well as osteoblasts. Filopodia are actin-based cell protrusions, which are regarded as one of the most important cellular sensors. They are used to collect space information and for sensing the substrate to determine areas suitable for cell attachment and proliferation, cell-cell interaction and allow cell migration toward the destination [59]. Therefore, formation of multiple filopodia, together with other presented results, clearly demonstrate the biocompatible properties of the tested nanomaterials.

## 5. Conclusions

The results of our works proved that amorphous titania nanotube coatings, used to modify the surface of medical devices made of titanium alloys, requires the removing of water remains on/inside nanotubular surface before autoclaving procedure—if we want, of course, the nanotube architecture remains on the surface. Additional drying affects the TiO_2_ tubes surface stabilization, making them resistant to the effects of hot water vapors under higher pressure, which are present during the sterilization process made by autoclaving. Otherwise, nanotubular coatings containing traces of water on the surface or inside the nanotubes during autoclaving can be subjected to the destruction of their tubular architecture and to promoting of amorphous TiO_2_ tubes phase transformation into polymorphic nanocrystals or crystalline powders. Such coatings revealed different mechanical and biointegration properties compared to nanotubular ones. 

## Figures and Tables

**Table 1:** Mechanical and nanoindentation properties (hardness, Young’s modulus and maximum depth of indentation) of reference Ti6Al4V, Ti6Al4V/TNH20-60 and Ti6Al4V/TNT20-60 systems.

Biomaterial Sample	Hardness (GPa)	Young’s Modulus (GPa)	Maximum Depth of Indentation (nm)
Ti6Al4V	16.17 ± 3.61	269.74 ± 40.10	162.14 ± 14.95
TNH20	10.24 ± 2.59	293.01 ± 59.43	194.40 ± 24.46
TNH30	9.15 ± 3.19	258.82 ± 57.44	212.80 ± 46.42
TNH40	4.95 ± 2.78	213.74 ± 87.35	323.33 ± 141.30
TNH50	4.43 ± 2.00	192.45 ± 56.42	312.17 ± 78.23
TNH60	6.51 ± 2.80	214.97 ± 52.72	258.13 ± 68.94
TNT20	19.38 ± 6.13	462.76 ± 245.91	143.99 ± 22.26
TNT30	16.81 ± 5.80	370.23 ± 109.44	160.08 ± 38.65
TNT40	9.42 ± 4.12	269.16 ± 79.77	212.39 ± 42.11
TNT50	9.56 ± 5.12	269.14 ± 91.83	217.36 ± 51.49
TNT60	14.32 ± 4.29	320.72 ± 77.26	169.28 ± 26.80

**Table 2:** Results of nano scratch-tests of Ti6Al4V/TNH20-60 and Ti6Al4V/TNT20-60 systems.

	Nanoscratch-Test Properties	
Coating	Critical Load (mN)	Critical Friction (mN)
TNH20	234.86 ± 53.53	266.87 ± 59.73
TNH30	254.14 ± 53.89	284.31 ± 73.77
TNH40	293.23 ± 54.71	355.05 ± 73.27
TNH50	268.78 ±83.19	316.54 ± 98.03
TNH60	241.61 ± 68.00	246.25 ± 84.18
TNT20	286.51 ± 77.35	307.92 ± 90.38
TNT30	336.65 ± 41.21	397.86 ± 79.63
TNT40	379.08 ± 46.38	417.66 ± 68.00
TNT50	353.01 ± 12.82	388.39 ± 17.87
TNT60	271.52 ± 46.79	311.04 ± 66.94

## Supplementary Materials

The following are available online at https://www.mdpi.com/2077-0383/8/2/272/s1, Figure S1: IR DRIFT spectra of TNH20-TNH60; Figure S2: The values of contact angles for water (a) and diiodomethane (b), and surface free energy (c) of Ti6Al4V/TNT20-60 and Ti6Al4V/TNH20-60 samples; Table S1: Diameters and wall thickness of titania nanotubes produced on the surface of Ti6Al4V substrates in the potential range of 5–60V; Table S2: Contact angles values for Ti6Al4V/TNT20-60 and Ti6Al4V/TNH20-60, measured for water and diiodomethane, and surface free energy values obtained according to Owens-Wendt method; Table S3: Surface roughness parameters (S_a_) of Ti6Al4V, Ti6Al4V/TNT20-60 and Ti6Al4V/TNH20-60 systems, as determined based on the AFM image analysis.
