# Supplementary material for: The Morphology, Structure, Mechanical Properties and Biocompatibility of Nanotubular Titania Coatings before and after Autoclaving Process
Source: J Clin Med. 2019 Feb 23;8(2):272. doi: 10.3390/jcm8020272 (PMC6406720; doi:10.3390/jcm8020272)
Supplement: Supplementary file 1 [file jcm-08-00272-s001.pdf]

Journal of
Clinical Medicine

MDPI

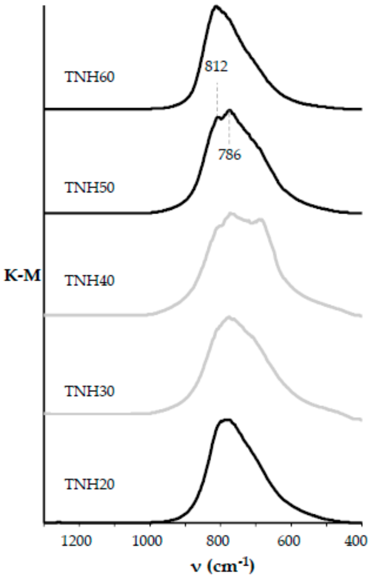

Figure S1. IR DRIFT spectra of TNH20-TNH60.

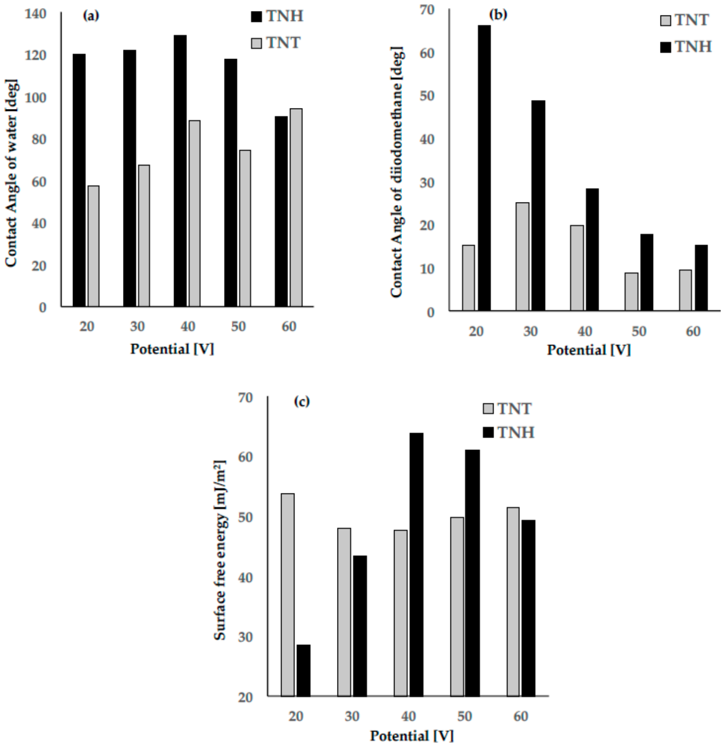

Figure S2. The values of contact angles for water (a) and diiodomethane (b), and surface free energy (c) of Ti6Al4V/TNT20-60 and Ti6Al4V/TNH20-60 samples.

Table S1. Diameters and wall thickness of titania nanotubes produced on the surface of Ti6Al4V substrates in the potential range of 5–60 V.

Sample	Potential (V)	Tubes diameter (nm)	Wall thickness (nm)
TNT5	5	25-35	4-5
TNT10	10	30-45	4-5
TNT15	15	50-70	c.a. 6
TNT20	20	65-90	c.a. 9
TNT30	30	90-150	c.a. 15
TNT40	40	100-250	c.a. 15
TNT50	50	80-240	c.a. 14
TNT60	60	30-110	c.a. 10

Table S2. Contact angles values for Ti6Al4V/TNT20-60 and Ti6Al4V/TNH20-60, measured for water and diiodomethane, and surface free energy values obtained according to Owens-Wendt method.

Biomaterial sample	Average contact angle [°] ± standard deviation		Surface free energy ± standard deviation [mJ/m²]
	Measuring liquid		
	Water	Diiodomethane	
TNT20	57,1 ± 0,90	15,1 ± 0,17	53,70 ± 0,14
TNT30	67,1 ± 0,60	25,1 ± 1,40	48,00 ± 0,47
TNT40	88,5 ± 1,91	19,8 ± 1,53	47,56 ± 0,49
TNT50	74,00 ± 0,90	8,8 ± 0,16	49,83 ± 0,11
TNT60	94,1 ± 0,55	9,4 ± 1,25	51,38 ± 0,37
TNH20	119,8 ± 0,07	66,1 ± 0,01	28,4 ± 0,00
TNH30	121,6 ± 0,12	48,7 ± 1,77	43,39 ± 0,53
TNH40	129,1 ± 0,07	28,2 ± 0,28	63,76 ± 0,11
TNH50	117,5 ± 0,95	17,7 ± 0,48	60,97 ± 0,21
TNH60	90,3 ± 0,11	15,2 ± 1,20	49,18 ± 0,36

Table S3. Surface roughness parameters (S_a) of Ti6Al4V, Ti6Al4V/TNT20-60 and Ti6Al4V/TNH20-60 systems, as determined based on the AFM image analysis.

Reference Sample	S_a parameter [μm]	Ti6Al4V/TNH Samples	S_a parameter [μm]	Ti6Al4V/TNT Samples	S_a parameter [μm]
Ti6Al4V	0.027	TNH20	0.075	TNT20	0.058
		TNH30	0.076	TNT30	0.065
		TNH40	0.110	TNT40	0.102
		TNH50	0.189	TNT50	0.131
		TNH60	0.172	TNT60	0.081
